# Stress Hyperglycemia, Diabetes Mellitus and COVID-19 Infection: Risk Factors, Clinical Outcomes and Post-Discharge Implications

**DOI:** 10.3389/fcdhc.2022.826006

**Published:** 2022-04-07

**Authors:** Antonina Gerganova, Yavor Assyov, Zdravko Kamenov

**Affiliations:** ^1^Department of Internal Medicine, Medical University - Sofia, Sofia, Bulgaria; ^2^Clinic of Endocrinology, University Hospital Alexandrovska, Sofia, Bulgaria

**Keywords:** COVID-19, new-onset hyperglycemia, new-onset diabetes, post-discharge, SARS-CoV-2

## Abstract

The novel severe acute respiratory distress syndrome-coronavirus 2 (SARS-CoV-2) has caused one of the most substantial pandemics that has affected humanity in the last century. At the time of the preparation of this review, it has caused the death of around 5 million people around the globe. There is ample evidence linking higher mortality risk rates from Coronavirus disease-19 (COVID-19) with male gender, advancing age and comorbidities, such as obesity, arterial hypertension, cardiovascular disease, chronic obstructive pulmonary disease, diabetes mellitus, and cancer. Hyperglycemia has been found to be accompanying COVID-19 not only in individuals with overt diabetes. Many authors claim that blood glucose levels should also be monitored in non-diabetic patients; moreover, it has been confirmed that hyperglycemia worsens the prognosis even without pre-existing diabetes. The pathophysiological mechanisms behind this phenomenon are complex, remain controversial, and are poorly understood. Hyperglycemia in the setting of COVID-19 could be a consequence of deterioration in pre-existing diabetes, new-onset diabetes, stress-induced or iatrogenic due to substantial usage of corticosteroids within the context of a severe COVID-19 infection. It is also plausible that it might be a result of adipose tissue dysfunction and insulin resistance. Last but not least, SARS-CoV-2 is also claimed to trigger sporadically direct β-cell destruction and β-cell autoimmunity. Pending further validations with longitudinal data are needed to legitimize COVID-19 as a potential risk factor for the development of diabetes. Hereby, we present an emphasized critical review of the available clinical data in an attempt to unravel the complex mechanisms behind hyperglycemia in COVID-19 infection. The secondary endpoint was to evaluate the bidirectional relationship between COVID-19 and diabetes mellitus. As the worldwide pandemic is still expanding, demand for answering these questions is arising. It will be of immense help for the management of COVID-19 patients, as well as for the implementation of post-discharge policies for patients with a high risk of developing diabetes.

## Introduction

The novel SARS-CoV-2 has caused one of the most substantial pandemics that has affected humanity in the last century. At the time of compiling this present review, globally, it has caused the death of around 5 million people (1). Its rapid spread has attracted medical specialists’ attention to its relationship with other common comorbidities such as obesity, arterial hypertension, cardiovascular disease, chronic obstructive pulmonary disease, diabetes mellitus (DM) and cancer (2–6).

Diabetes, particularly type 2 diabetes mellitus (T2DM), has been reported as the second most common comorbidity of COVID-19 after hypertension (7–12). There is burgeoning evidence that comorbidities increase the morbidity and mortality in SARS-CoV-2 infection and that the DM patients are frequently found to have severe infection (13–16). However, the data regarding outcomes classified according to glycemic control is scarce. A couple of studies have demonstrated worse outcomes in patients with poor-controlled diabetes (17–19). Additionally, several pieces of research have demonstrated that pre-existing diabetes, newly detected diabetes, prediabetes, and uncontrolled hyperglycemia are significant determinants of COVID-19 prognosis (20–24). However, hyperglycemia has been found to be accompanying COVID-19 not only in patients with pre-existing diabetes. Hereby, we present an emphasized critical review of the available clinical data in an attempt to elucidate the complex mechanisms behind hyperglycemia in COVID-19. The secondary endpoint was to evaluate the bidirectional relationship between COVID-19 and diabetes mellitus.

The presence of diabetes in COVID-19 patients has been frequently established, and diabetes patients are a known high-risk group in COVID-19 disease (21, 25, 26). Furthermore, an abrupt increase of plasma glucose regardless of prior diabetes and acute deterioration in the glycemic control of pre-existing diabetes in the setting of COVID-19 is found to be a common case scenario. These findings suggest a bidirectional relationship between stress-induced hyperglycemia and COVID-19.

The CoviDIAB Project has been started by leading diabetes professionals and is dedicated to establishing and managing a global registry of patients with COVID-19-related diabetes (5, 27). However, a growing body of literature suggests that ‘‘new-onset” hyperglycemia is a common phenomenon at hospital admission of COVID-19 patients, who had neither prior history of dysglycemia or diabetes nor current corticosteroid administration (28). It could be categorized as – 1.) “stress hyperglycemia”; 2.) “new-onset diabetes” in previously unrecognized dysglycemia; 3.) hyperglycemia, associated with SARS-CoV-2 direct impact on endocrine system; and 4.) in-hospital hyperglycemia due to glucose-altering medications such as glucocorticosteroids, etc. (28). This new-onset hyperglycemia was found by many authors to be an independent predictor for mortality and would be of particular interest to this scientific review (28, 29).

## Stress Hyperglycemia

Stress hyperglycemia is most commonly defined as hyperglycemia that spontaneously resolves after overcoming a critical condition (30, 31). This expression addresses patients predominantly without prior diabetes. However, some people with diabetes could also develop stress hyperglycemia. This often remains unnoticed in many studies comparing in-patients with or without diabetes (30, 32–34). Transient elevation of blood glucose has been thought to be unhazardous or even beneficial (31, 35, 36). No guideline specifically defines its cut-offs. The Diabetes in Hospitals Writing Committee of the American Diabetes Association (ADA) has published a report in which in-hospital hyperglycemia is classified into known diabetes, newly diagnosed diabetes, and hospital-related hyperglycemia (37).

Furthermore, Dungan et al. (2009) proposed two types of stress hyperglycemia – hospital-related hyperglycemia in concordance with the ADA consensus definition and deterioration of glycemic control in pre-existing diabetes (30). They also suggested that the most suitable cut-off for stress hyperglycemia in diabetic patients has to be clarified. However, a patient with glycosylated hemoglobin (HbA1c) within target (<7%), whose blood glucose levels are steadily more elevated than the cut-off defined for hospital-related hyperglycemia, would also qualify (30). In North America, one-third of people with diabetes are unaware of their clinical condition (38). Thus, many patients presenting at hospital admission with stress hyperglycemia could have had a pre-existing carbohydrate disturbance (30, 39–45).

The three major pathways for the development of stress hyperglycemia are:

1. Increased hepatic gluconeogenesis by means of elevated contrainsulatory hormones
2. Peripheral insulin resistance
3. Beta-cell dedifferentiation

The illness may impact the scale of cytokine production and hormonal imbalance, which could lead to excessive hepatic glucose output, mainly *via* gluconeogenesis and insulin resistance (IR) (46, 47). Gluconeogenesis is primarily induced by glucagon, but epinephrine and cortisol also contribute (48–50). During illness, the observed insulin resistance is mainly due to the inability of insulin to inhibit liver gluconeogenesis (30).

Peripheral insulin resistance is a consequence of defects in post-receptor insulin signaling and downregulation of glucose transporter (GLUT)-4 (30, 51). Furthermore, epinephrine also leads to insulin receptor phosphorylation and impedes its tyrosine kinase activity, thus causing immediate and protracted inhibition of pancreatic insulin secretion (52, 53). Cytokines such as TNFα and interleukin 1 inhibit post-receptor insulin signaling (48, 54). The more severe the condition is, the more considerable increase in cytokines levels and insulin resistance is observed (55, 56). Glucotoxicity in the context of an acute state is facilitated by upregulated GLUT-1 and GLUT-3 transporters, allowing uncontrolled glucose cell entering despite downregulation mechanisms (57, 58).

Finally, hyperglycemia is presumed to be the significant determinant causing β-cells to lose differentiation, resulting in dysfunctional insulin secretion (59). Some authors also suggest that stress hyperglycemia indicates relative insulin deficiency attributed to enhanced lipolysis and elevated circulating free fatty acids, observed in the acute state (60). Glucotoxicity, lipotoxicity, and inflammation are significant contributors to the global insulin-resistance syndrome in critical illness (30).

### SARS-CoV-2 infection and the pancreas

As previously established, DM was found to be an independent predictor of unsatisfactory outcomes even during previous coronavirus outbreaks (61, 62). Moreover, acute diabetes was frequently encountered during the SARS-COV-1 epidemic in individuals who had neither a prior history of diabetes nor any glucocorticoid administration; furthermore, it was an independent prognostic factor for mortality (61). The pathophysiological mechanism behind the sudden spike of plasma glucose levels was confirmed to be a result of massive pancreatic islet damage, following the docking of SARS-CoV-1 to angiotensin-converting enzyme 2 (ACE2) receptor (63). Additionally, Niu et al. (2008) proved that ACE2 knockout experimental animals develop acute diabetes (64, 65).

There is burgeoning evidence of ACE2 expression in multiple tissues throughout the body, namely intestines, kidneys, myocardium, vasculature. However, pancreatic ACE2 expression is of particular interest due to a rising number of reports pointing out a surge in patients with new-onset hyperglycemia and ketoacidosis and giving rise to questions about whether COVID-19 actually induces diabetes *via* β-cell injury (27, 66–70). A certain number of study groups have investigated non-diabetic, diabetic, and COVID-19 pancreatic tissue samples for the expression of various entry factors in assessing SARS-CoV-2 diabetogenic potential (71, 72). Most of them concur that ACE2 and transmembrane protease, serine 2 - TMPRSS2 proteins are established in pancreatic ducts and microvasculature endothelial cells, which could promote indirect impairment of pancreatic endocrine function in COVID-19 (73). Nevertheless, comprehensive data regarding ACE2 and TMPRSS2 expression in exocrine cells remain discrepant because researches that discover entry factors outside β-cells do not identify SARS-CoV-2 nucleocapsid protein in COVID-19 pancreas tissues (71, 72).

Conversely, some authors claim that after endocytosis of the virus complex, ACE2 expression is downregulated, acting dually. To begin with, this may provoke islet function impairment, leading to β-cell damage (65). Secondly, its downregulation causes uncontested angiotensin II action, which could impede following insulin secretion by blood flow restriction and increase of oxidative stress in β-cells (65). Additionally, aberrant glycosylation of the ACE2 receptor fosters the consolidation of the SARS-CoV-2 virus and the ACE2 receptor, thereby worsening COVID-19 severity (74–76). This finding could be induced by hyperglycemia, pointing to a vicious cycle. A recent report documented that hyperglycemia permits SARS-CoV-2 replication and ACE2 expression in monocytes accumulated in the lung of COVID-19 patients, thus prompting mitochondrial reactive oxygen species (ROS) production by stabilizing hypoxia-inducible factor-1a (HIF-1a) and promoting glycolysis (77). These observations could partially elucidate the greater propensity of hyperglycemic and diabetic patients for SARS-CoV-2 infection and severe illness (78, 79).

Interestingly, a recent study by Steenblock et al. (2021) demonstrated in cadavers that 70% of the COVID-19 patients have vasculature ACE2 expression, but just 30% showed ACE2-expression in insulin-producing islet cells. Even when new-onset diabetes is not present, necroptotic cell death, immune cell infiltration, and SARS-CoV-2 viral infection of pancreatic β-cells may promote metabolic imbalance in COVID-19 individuals (80). Utilizing human islets and cadaver pancreatic samples from patients that died of COVID-19, they clearly demonstrated that β-cells are permissive to infection with SARS-CoV-2 (80). However, the mechanisms of virus entry are not totally understood so far, as β-cells ACE2 expression is not detected in all patients. Hence, other factors may facilitate the uptake of SARS-CoV-2 into β-cells (80). It seems that the answer to the question “How SARS-CoV-2 induces hyperglycemia?” is not straightforward, and there could be not only one correct answer.

In an attempt to unravel that question, Clark and Mirmira (2021) discuss the current evidence and implications in SARS-CoV-2 infection of islet β-cells (81). Two of the commented studies were lately presented by Wu et al. (2021) and Tang et al. (2021) (81–83). In the study of Wu et al. (2021), the authors verify the previously established low levels of ACE2 and TMPRSS2 in β-cells but propose other entry factors, such as NRP1 and TRFC which may serve as viral entry points (82, 84–86). The authors postulated that pancreatic endocrine dysfunction with decreased insulin secretion in response to glucose might be related to the viral SARS-CoV-2 invasion (81, 82). Remarkably, insulin-producing cells self-destruction mechanism was found to be also involved, as a result of signaling and further triggering of the mitogen-activated protein kinase (MAPK)/c-Jun N-terminal kinase (JNK) pathways. Researchers ruled out that inhibition of NRP1 prevents an additional mechanism for SARS-CoV-2 to achieve cell invasion (81, 82).

Furthermore, Tang et al. (2021) indicate the presence of the same entry factors — ACE2 and NRP1 — in human β-cells and prove that SARS-CoV-2 is able to infect them *in vitro* (83). They also demonstrate in infected cells that the reduction in insulin levels is followed by an increase in glucagon (a typical feature of α-cells) and trypsin1 (a typical feature of exocrine cells) and that upon infection, β-cells underwent eIF2-mediated transdifferentiation (83). However, these studies have some limitations. Namely, both were performed utilizing human islets infected *in vitro*, and it is not clear whether their findings are valid *in vivo* in COVID-19 individuals. Even though both provided conclusive proof of viral antigens in COVID-19 cadavers, the feasibility that this is actually absorbed viral debris still remains. Moreover, the probability that *in vitro* infectivity might be limited to certain people is also durable.

Collectively, the results from Wu et al. (2021), Tang et al. (2021) and a recent study by Muller et al. (2021) emphasize on several controversial key points in regards to diabetes pathogenesis in the context of COVID-19 infection (82, 83, 87). It was suggested for the first time that the viral infection with SARS-CoV-2 could trigger an autoimmune process against β-cells (82, 83, 87). Studies suggesting an extensive amount of entry factors in β-cells not only identify SARS-CoV-2 nucleocapsid protein in COVID-19 pancreatic tissues but show that human islets could be infected with SARS-CoV-2 ex vivo to disorganize insulin homeostasis and provoke β-cell apoptosis. The latter could result in extensive pathology that could drive T1D-associated hyperglycemia (71, 72). This theory is based on virus-mediated damage of β-cells and release of hitherto sequestered antigens that cause the activation of autoreactive T-lymphocytes, resulting in an autoimmune response that significantly damages the β-cell remnant (88).

Moreover, Qadir et al. (2021) recently reported pancreatic fibrosis and thrombosis in new-onset diabetes in humans and primates with COVID-19 (89). A cytokine storm in COVID-19 patients is a prothrombotic, highly inflammatory pathological state that is able to, directly and indirectly, affect β-cells (90). It is presumed that in the context of COVID-19, stress hyperglycemia could be more pronounced on account of the cytokine storm (90). Another study demonstrated in three cadavers, who died from COVID-19, degeneration of pancreatic islets (91). Research from Wuhan (n=121) found that individuals with mild COVID-19 had elevated pancreatic enzymes (1.85%), but those with severe COVID-19 had much greater levels (17%) (92). A part of them also was symptomatic of acute pancreatitis. However, despite these findings, no evident tendency of increase in type 1 DM incidence during the pandemic has been documented (81, 93). Moreover, an Indian longitudinal study investigated the deterioration of β-cell function, insulin resistance and glycemic progression and did not prove any of them (94). However, the authors enrolled mainly mild/asymptomatic SARS-CoV-2 patients, which could potentially bias the experimental findings (94). Pending further validations with longitudinal data are needed to legitimize COVID-19 effect on type 1 and type 2 diabetes development. Moreover, additional data is required to assess the subset of patients that potentially develop COVID-19 induced diabetes, their risk and predisposing factors.

### SARS-CoV-2 infection and the adipose tissue

Adipose tissue (AT) has been revealed to have upregulated ACE2 receptor activity making it a target for SARS-CoV-2 invasion. Obesity and advancing age are often accompanying comorbidities of T2DM and IR, that are highly linked to severe COVID-19 (2, 3, 5). They are also associated with visceral AT enlargement which induces low-grade inflammation (95). AT produces inflammatory adipokines and cytokines that regulate blood sugar levels and IR; inflammatory T2DM agents, including TNF-α, IL-6, MCP-1, and angiotensin, which are increased in critically ill COVID-19 individuals (95, 96). COVID-19 could adversely impact adipocytes and worsen chronic low-grade inflammatory state which deteriorates IR, elevated blood sugar levels and outcomes in SARS-CoV-2 infected DM patients (73). Individuals with SARS-CoV-2 infection and uncontrolled glycemia have higher concentrations of inflammatory biomarkers than patients without diabetes, including C-reactive protein (CRP), ferritin, and IL-6 (17). Only one report so far documented SARS-CoV-2 in 62.5% of postmortem ATs and detected nucleocapsid protein surrounding the cytoplasm of lipid droplets (97). Unfortunately, they did not estimate it (97).

In addition, Reiterer et al. (2021) found that among 4,102 US hospitalized COVID-19 patients, those with acute respiratory distress syndrome (ARDS) had a higher prevalence of hyperglycemia with poor outcomes (85%) than those without ARDS (37%) (98). They also report that serum levels of C-peptide and amylin were increased in COVID-19 patients with ARDS, indicative of β-cell hypersecretion that is inconsistent with the theory of widespread β-cell failure in COVID-19 (98). SARS-CoV-2 infected patients with ARDS (62% of them had no prior diabetes history) also had high C-peptide–to–glucose ratios, supporting rates of IR that were three-to-six fold higher than those of the control group (98). Additionally, a reduction of serum adiponectin levels by 50–60% was observed. In contrast, leptin was increased in COVID-19 patients with ARDS, resulting in adiponectin-to-leptin ratios that would support AT dysfunction in IR (98). Similarly, Ceriello et al. (2020) and Apicella et al. (2020) proposed insulin resistance and possibly insulin secretory abnormalities, which could precipitate hyperglycemia in patients with COVID-19, even in the absence of pre-existing diabetes (99, 100).

In keeping with these findings, the study of Montefusco et al. (2021) merits a mention. They investigated long-term glucose homeostasis deterioration after acute SARS-CoV-2 infection (101). In 253 out of 551 hospitalized Italian patients (46%) with no prior diabetes history, new-onset hyperglycemia was established during acute COVID-19. Among them, 35% still had hyperglycemia 6 months after COVID-19 recovery, while an additional 2% were found to have T2DM, suggesting that new-onset elevation of blood glucose can prompt patients to long-term glycemic abnormalities (101). In accordance with the results of Reiterer et al. (2021), patients with T2DM, those with acute COVID-19, and those who recovered from COVID-19 all were found to yield greater insulin and C-peptide secretion following arginine administration, consistent with acute and long-term β-cell hypersecretion and IR following COVID-19 (98, 101). More extensive studies of such kind are necessary to verify whether these long-term abnormalities change new-onset diabetes incidence rates.

## Metabolic Outcomes of COVID-19

The impact of stress hyperglycemia on the clinical outcomes of COVID-19 in-patients has been thoroughly studied. COVID-19, like any other viral infection, induces a stress reaction. However, there is no likelihood that it can influence the HbA1c but could potentially elevate blood glucose levels. Thus, an isolated fasting plasma glucose (FPG) value of ≥ 7.0 mmol/L in the presence of HbA1c < 6.5% has been classified by some researchers as new-onset hyperglycemia without diabetes. It is important to highlight that the latter term easily fit both “stress hyperglycemia” and “new-onset diabetes” in previously unrecognized dysglycemia.

Similarly to Singh, Singh (2021), for the sake of clarity, we stratified the available data so far about COVID-19 outcomes and carbohydrate disturbances into four categories: a. new-onset hyperglycemia without diabetes versus normoglycemia; b. new-onset hyperglycemia without diabetes versus diabetes (new-onset and pre-existing); c. new-onset diabetes versus normoglycemic patients; and d. new-onset diabetes versus pre-existent (28).

### New-Onset Hyperglycemia Without Diabetes Versus Normoglycemia

Firstly, Bode et al. (2020) focused the attention on stress hyperglycemia in COVID-19 patients. They established that elevated plasma glucose in people with DM (HbA1c ≥ 6.5%) or uncontrolled hyperglycemia without prior diabetes was related to an increase in mortality in comparison to normoglycemic subjects (28.8% vs 6.2% respectively; p < 0.001) (20). Uncontrolled hyperglycemia was defined as two or more blood glucose measurements > 10,0 mmol/l occurring within any 24-hour period with an HbA1c < 6.5%, or no HbA1c testing done during hospitalization (20). Zhang et al. (2020) showed a 5-fold increase in composite outcome risk (mechanical ventilation [MV], admission in intensive care unit [ICU] and death) in people with a secondary elevation of plasma blood glucose (defined as FPG ≥ 7.0 mmol/L before glucocorticoid administration, but HbA1c < 6.5%) and COVID-19, in comparison with normoglycemic patients (102). Hereinafter for all of the studies and their respective results, the composite outcome should mean MV, ICU admission and death.

Concurrently, in severe COVID-19 individuals, a 71% relative mortality risk reduction was demonstrated for individuals with normal blood glucose levels (with or without diabetes) as opposed to those with at-admission hyperglycemia (new-onset hyperglycemia without diabetes or pre-existing diabetes, with FPG > 7.77 mmol/l) (103). Additionally, Mamtani et al. (2020) retrospectively reported in 403 COVID-19 patients that the prevalence of hyperglycemia was 56.6%, utilizing the cut-off value of > 7,78 mmol/l (21). This finding is slightly higher than the prevalence reported in non-COVID-19 in-patients - 38–40% (21, 104). They also have found that hyperglycemic hospitalized non-diabetic COVID-19 patients as a subgroup (20.6%) are associated with higher mortality risk and poor clinical outcomes (21). They implied that hyperglycemia within the first 48 hours of admission could be used as an independent predictor of COVID-19 prognosis, and early stress hyperglycemia in non-diabetic patients could indicate increased systemic stress (21). They even conjectured that hyperglycemia might contribute to the development of cytokine storm (21).

Likewise, Wang et al. (2020) observed more than double increase in the 28-day in-hospital complication rate of hyperglycemic non-diabetic COVID-19 subjects (FBG 6.1–6.9 mmol/L), in comparison to normoglycemic ones (22). At the same time, Li et al. (2020) confirmed a negative tendency in all-cause mortality (HR 2.64; 95% CI, 0.50–14.0) at a 30-day-follow-up in hyperglycemic patients without DM (FPG 5.6–6.9 mmol/L and/or HbA1c 5.7–6.4%), contrary to those with normal plasma glucose (FPG < 5.6 mmol/L and HbA1c < 5.7%) (23). Moreover, Coppelli et al. (2020) found that mortality was substantially increased in hyperglycemic individuals without diabetes (defined as no prior diabetes and FPG ≥ 7.78 mmol/L at admission) in contrast to normoglycemic COVID-19 individuals (at-admission blood glucose < 7.78 mmol/L) - 39.4% vs 16.8% respectively; HR 2.20; 95% CI, 1.27–3.81; p = 0.005 (105). In their study, 21% had DM (n=271), and slightly more (24%) had at-admission glycemia ≥ 7.78 mmol/L. There was no one with new-onset hyperglycemia who had a prior DM diagnosis (105). All of them were not taking any glucose-altering medication, supporting the recent development of hyperglycemia (105).

In one of the most recently published studies, Haymana et al. (2021) performed a retrospective analysis of 12,817 non-diabetic COVID-19 patients that were stratified in regards to their blood glucose levels, as follows: group 1 - < 5,5 mmol/l; group 2 - 5,5 – 7,7 mmol/l and group 3 – 7,8 – 11,0 mmol/l (7). They recorded plasma blood glucose measurements within 24 hours of COVID-19 diagnosis regardless of fasting state. Patients in group 2 (5%) and group 3 (14%) were found to have higher mortality rates than group 1 (2,1%). Furthermore, glucose levels in the range of 7,8 - 11,0 mmol/l were an independent associate of mortality (2.7 fold increased risk compared to normoglycemia) and the composite of ICU admission and/or MV (2.3 fold increased risk compared to normoglycemia) (7). Similarly, Ilias et al. (2021) documented that both COVID-19 patients in the wards and in the ICU may manifest with higher-than-expected glycemia, even in the absence of diabetes (86% without prior history of diabetes) (106). Their findings lend credence to suggestions of compromised insulin secretion and lowered sensitivity to insulin in COVID-19 patients (106).

In summary, most of the aforementioned studies were performed in the initial stages of the global COVID-19 pandemic and/or investigated glucose levels at hospital admission. Their results are not potentially biased by any glucose-altering medications and unequivocally demonstrate that stress hyperglycemia/new-onset hyperglycemia without diabetes in COVID-19 patients is related to increased mortality risk. The risk is even higher in patients without prior DM, as confirmed by the meta-analysis of Lazarus et al. (2020). They demonstrated 75% increased risk for poor outcome in patients without history of DM (107). However, each of the studies has its own cut-off value of elevated blood glucose, which should be considered as a significant confounding factor.

### New-Onset Hyperglycemia Without Diabetes Versus Diabetes (New-Onset and Pre-Existing)

Firstly, Bode et al. (2020) among 1122 patients acknowledged an increase in mortality of COVID-19 individuals with new-onset hyperglycemia without diabetes, in comparison to those with pre-existing DM (41.7% vs 14.8% respectively; p < 0.001) (20). Moreover, Zhang et al. (2020) reported that the composite risks for new-onset hyperglycemia (FBG ≥ 7.0 mmol/L once or HbA1c < 6.5%) and new-onset diabetes were as follows 5,47 and 2,61 (102). Supposedly, it was one of the first studies that recommended clinicians to pay close attention to blood glucose levels in COVID-19 patients, even in those without prior diabetes. Conversely, they revealed an increasing tendency, however not statistically significant, in composite outcomes between patients with new-onset hyperglycemia without diabetes and with diabetes (new-onset or pre-existing).

### New-Onset Diabetes Versus Normoglycemic Patients

Regarding the outcome of COVID-19 patients with new-onset diabetes versus normoglycemic ones, new data is constantly emerging. Firstly, Zhang et al. (2020), in their retrospectively enrolled 166 COVID-19 patients from Wuhan, established new-onset diabetes (FBG ≥ 7.0 mmol/L twice before glucocorticoid therapy administration or HbA1c ≥ 6.5%) in 16% of the cases (26/166) (102). In the new-onset diabetes group, the authors highlighted that they prioritized the results of HbA1c over FPG in the grouping criteria to exclude the possibility of overestimating the incidence of diabetes (102). Notably, no significant increase in composite outcomes risk was observed in diabetic patients (both new-onset and pre-existing) in comparison to SARS-CoV-2 infected individuals with normal blood glucose (102). Li et al. (202) retrospectively analyzed 453 patients and demonstrated incidence of new-onset diabetes (FPG ≥ 7 mmol/L and/or HbA1c ≥ 6.5%) corresponding to 21% (n=94) (23). They demonstrated a significant increase (30 days mean follow-up) in all-cause mortality (HR 5.63; 95% CI, 1.22–26.0) in comparison with normoglycemic COVID-19 patients (23). Likewise, Wang et al. (2020) reported similar incidence of new-onset diabetes (FBG ≥ 7.0 mmol/L) - 29% of cases (176/605) with statistically significant complication rate (OR 3.99; 95% CI, 2.71–5.88) and all-cause mortality (HR 2.30; 95% CI, 1.49–3.55; p = 0.002), contrary to COVID-19 patients with normal blood glucose (22). Yang et al. (2020) retrospectively reviewed 120 patients and found that 69 had new-onset diabetes (108). New-onset diabetes (FBG ≥ 7.0 mmol/L for two times during hospitalization) was demonstrated as an independent predictor for death (HR 3.75; 95% CI 1.26–11.15; p = 0.017) even after a multivariable analysis (108). However, it seems that both cases with secondary hyperglycemia and new-onset diabetes in the last three papers fit the criteria for new-onset diabetes. It is important to note that in all of them, individuals on glucocorticoid therapy were excluded.

Another research from last year of Fadini et al. (2020) on the contrary showed that 5% out of 413 had new-onset diabetes (HbA1c ≥ 6.5% or a random glucose level ≥ 11.1 mmol/L with symptoms of elevated blood glucose) (67). There was a substantial elevation (RR 3.06; 95% CI, 2.04–4.57) in severe COVID-19 rates (ICU admission and death) in people with new-onset diabetes, as opposed to normoglycemic individuals (67). Last but not least, Sun et al. (2021) retrospectively analyzed a total of 268 COVID-19 patients; 19,3% of those with comorbidities had diabetes (n=21) (9). The study yielded interesting results that could possibly associate severe SARS-CoV-2 infection in patients with present clinical laboratory findings of serum glucose levels ranging from normal (5.53 mmol/l) to slightly elevated (7.27 mmol/l) (9). The authors’ collective noticed better survival rates in patients with plasma glucose levels < 5.53 mmol/l than in individuals with laboratory findings over the previously mentioned cut off. A possible pathophysiological link was proposed to be present between the severe course of the disease, the abnormal blood glucose levels and the unfavorable outcome (9). Therefore, there might be a rationale behind the necessity for tight supervision of elevated blood glucose in pneumonia cases (9).

In keeping with these results, some latest meta-analyses and studies showed sufficient data regarding the topic (109–112). The first one demonstrated associated DM and hyperglycemia in 19.70% (CI: 10.93-32.91) and 25.23% (CI: 19.07-32.58) of COVID-19 cases, respectively (110). The observed mortality rate remained significantly higher (15.36%) in spite of their DM and its status. On the other hand, a decreased tendency of mortality rate has been confirmed in non-diabetic and patients with SARS-CoV-2 related hyperglycemia (110). Additionally, higher death rates and adverse events were observed in patients with new-onset DM and elevated plasma glucose than in the non-diabetic population (110). The second meta-analysis enrolled 9045 patients from 12 studies and reconfirmed that fasting hyperglycemia is related to mortality in COVID-19 patients, with or without diabetes (109).

### New-Onset Diabetes Versus Pre-Existent

So far, we are lacking extensive data comparing outcomes of patients with new-onset and pre-existing DM. As previously mentioned, Li et al. (2020), in the early stages of COVID-19 pandemic, reported a nearly 2-fold higher risk of all-cause mortality in patients with new-onset diabetes (fasting glucose ≥7 mmol/L and/or HbA1c ≥ 6.5%) - HR 9.42; 95% CI, 2.18–40.7, compared to pre-existing diabetes (HR 4.63; 95% CI 1.02–21.0) vs COVID-19 normoglycemic individuals (23). Concurrently, Fadini et al. (2020) also demonstrated a stronger association in ICU admission rate or death in people with new-onset diabetes (RR 3.06; 95% CI, 2.04–4.57) in comparison to individuals with a prior diagnosis of diabetes (RR 1.55, 95% CI 1.06–2.27) (67). Last but not least, there is a fascinating recent cross-sectional prospective study by Farag et al. (2021). They studied 570 COVID-19 patients and classified them as non-diabetic or newly discovered DM according to HbA1c and fasting insulin after exclusion of known DM cases (113). Interestingly, 77 patients were diagnosed with DM (13.5%); 12 (2.1%) - with pre-existing DM, 7 (1.2%) - with new-onset type 1 DM, and 58 (10.2%) - with new-onset T2DM. Moreover, COVID-19 was related to a new-onset of DM in 11.4% of all participants and expression of pre-existing DM in 2.1% of all participants, both related to severe COVID-19 (113). Elevated plasma glucose and the necessity for glucose-lowering medication remained in 73% of diabetic cases (46/63), whereas anti-diabetic treatment could be terminated in 17 patients (27%) (113). High blood glucose remained in all survivors with pre-existing DM (*n* = 9) and in 68,5% of survived patients with new-onset DM types I and II (*n* = 54) (113). Furthermore, the death rate within the COVID-19 patients was substantially increased among newly diagnosed DM than non-diabetic patients (18.2% vs 9.7%, *p* = 0.046) (113).

Analyzing the available data about SARS-CoV-2 and hyperglycemia, it is clear that new-onset hyperglycemia and new-onset diabetes are related to higher mortality risk. Additional data regarding the abovementioned studies are summarized in **Table 1**. It is evident that different cut-offs (slightly higher/slightly lower), which do not cover standard criteria for abnormal blood glucose, could lead to over/underestimation of the observed findings. More scientific data is necessary to define how COVID-19 actually impacts carbohydrate metabolism and whether this finding is transient or indicates actual diabetes.

**Table 1:** Summary of studies dedicated to new-onset hyperglycemia/new-onset diabetes.

Authors, year, country	Time frame of enrollment	N	Study design	Age, years, mean, SD	Hyperglycemiа cut-off	Outcome
**Bode et al. (2020), USA (** 20**)**	01.03 – 06.04.2020	1122	Diabetes and/or uncontrolled hyperglycemia vs. Absence of diabetes or uncontrolled hyperglycemia	65 (24-95) - group with DM and uncontrolled hyperglycemia; 61 (18 - 101) - group without DM and hyperglycemia	Uncontrolled hyperglycemia 2 measurements of BG ≥ 10,0 mmol/l within 24-hour period; Diabetes HbA1c ≥ 6,5%	Death occurred in 41,7% of patients with uncontrolled hyperglycemia vs. 14,8% of those with diabetes (p < 0.001) Increased mortality in the group with uncontrolled hyperglycemia/diabetes in comparison to subjects with normoglycemia (28.8% vs 6.2% respectively; p < 0.001)
**Zhang et al. (2020), China (** 102**)**	08.02 – 21.03.2020	166	Control group vs. Secondary hyperglycemia vs. Diabetes	62,7±14, 2	No diabetes history, FPG ≥ 7,0 mmol/L before glucocorticoid administration, but HbA1c < 6.5%	OR 5.47; 95% CI 1.51–19.82; p = 0.010 for composite outcome risk in people with secondary hyperglycemia and COVID-19 in comparison to normoglycemic patients OR 2.61; 95% CI 0.86–7.88; p = 0.09 for composite outcome risk in people with diabetes and COVID-19 in comparison to normoglycemic patients
**Sardu et al. (2020), Italy (** 103**)**	Since 20.02.2020 – till manuscript preparation	59	Normoglycemia vs. Hyperglycemia (18% of them with previous history of diabetes)	68,5±5,8 – hyperglycemia group; 66,6 ± 11,5 – normoglycemia group	FPG > 7,77 mmol/l	Relative mortality risk reduction was demonstrated for patients with no hyperglycemia HR 0.29; 95% CI, 0.08–0.96; p = 0.04
**Mamtani et al. (2020), USA** **(** 21**)**	15.03 – 03.05.2020	403	No-DM/no-HG vs. No-DM/ HG vs. DM/HG vs. DM/no-HG	54,9±13,55	FPG ≥ 7,78 mmol/l	Compared to the reference group (no-DM/no-HG), the no-DM/HG patients demonstrated increased mortality - adjusted OR 21.94 (95% CI 4.04–119.0), P < 0.001]; improved prediction of death (P = 0.01) and faster progression to death (P < 0.01). Compared to the reference group (no-DM/no-HG), the DM/HG patients showed increased mortality [OR 17.06 (95% CI 3.46–84.1), P < 0.001).
**Wang et al. (2020), China (** 22**)**	24.01 – 10.02.2020	605	Normoglycemia vs. IFG vs. Hyperglycemia	59	FBG 6,1–6,9 mmol/L and FPG ≥ 7,00 mmol/l	Group with at admission FBG <6.1 mmol/l vs. those with admission FBG ≥7.0 mmol/l - OR 3.99 (95% CI 2.71 - 5.88) for in-hospital complications Group with at admission FBG <6.1 mmol/l vs. those with 6.1– 6.9 mmol/l - OR 2.61 (95% CI 1.64 - 4.41) for higher levels of in-hospital complications
**Li et al. (2020), China (** 23**)**	22.01 – 17.03.2020	453	Normal glucose vs. hyperglycemia vs. newly diagnosed diabetes vs known diabetes	61	FPG 5,6–6,9 mmol/L and/or HbA1c 5,7–6,4% for hyperglycemia	All-cause mortality - HR 2.64; 95% CI, 0.50–14.0) at a 30-day-follow-up for hyperglycemic patients without DM vs normoglycemic patients* All-cause mortality - HR 5.63; 95% CI, 1.22–26.0) at a 30-day-follow-up for newly diagnosed diabetes without DM vs. normoglycemic patients*
**Coppelli et al. (2020), Italy (** 105**)**	20.03 – 30.04.2020	271	Normoglycemia vs. known diabetes vs. new-onset hyperglycemia	N/A	FPG ≥ 7,78 mmol/L	Increased mortality rates for hyperglycemic patients without diabetes in comparison to normoglycemic COVID-19 individuals - HR 2.20; 95% CI, 1.27–3.81; p = 0.005
**Haymana et al. (2021), Turkey (** 7**)**	11.03 – 30.05.2020	12817	Normoglycemia vs. BG of 5,5 – 7,7 mmol/l vs. BG of 7,8 – 11,0 mmol/l	Whole study population - 44 years; group 2 – 45; group 3 - 55	group 2 - 5,5 – 7,7 mmol/l and group 3 – 7,8 – 11,0 mmol/l	Increased death rates in group 2 (5%) and group 3 (14%) in contrast to group 1 (2,1%); p < 0,05 Increased mortality rates in group 2 in comparison to group 3; p < 0,05
**Ilias et al. (2021), Greece (** 106**)**	04 – 10.2020	157	No history of diabetes in the wards vs. History of diabetes in the wards vs. No history of diabetes in the ICU vs. History of diabetes in the ICU	60,2±15,3	No/History of diabetes	COVID-19 individuals without diabetes in the ICU had higher glucose than patients without diabetes in the wards (p= 0.0077)
**Fadini et al. (2020), Italy (** 67**)**	02 – 04.2020	413	Normoglycemia vs New-onset diabetes vs. Diabetes	64,9±15,4	Newly-diagnosed diabetes - HbA1c value of 6.5% or higher; in the absence of an HbA1c measurement, a RBG of 11,1 mmol/l or higher, accompanied by signs and symptoms of hyperglycemia.	Composite outcome risk for new-onset diabetes vs normoglycemia – OR 3,06 (2,04 – 4,57), p < 0,001
**Sun et al. (2021), China (** 9**)**	02.02 – 25.03.2020	268	Normoglycemia vs. patients with 5,53 – 7,27 mmol/l vs. subjects with BG ≥ 7,27 mmol/L	57,75	Group 2 - 5,53 to 7,27 mmol/L and group 3 - ≥ 7,27 mmol/L	Better survival rates for those with BG <5.53 mmol/L in contrast to those with BG ranging from 5.53 to 7.27 mmol/L (HR 6.34; 95% CI, 1.45-27.71) and ≥ 7.27 mmol/L (HR, 19.37; 95% CI, 4.68-80.17)
**Farag et al (2021), Egypt (** 113**)**	01.04 – 31.05.2020	570	Non-diabetic individuals vs. Newly discovered DM	47,9±10.9	Newly diagnosed DM - no preceding history of DM with FPG ≥ 7,0 mmol/l or RBG ≥ 11,1 mmol/l and HbA1c < 6.5%; newly discovered unrecognized DM FPG ≥ 7,0 mmol/l or RBG ≥ 11,1 mmol/l and HbA1c ≥ 6,5%	Higher mortality in patients with newly discovered DM in comparison to non-DM individuals (18,2% vs. 9,7%, p = 0,046 )

BG, blood glucose; FBG, fasting blood glucose; OR, odds ratio; CI, confidence interval; HR, hazard ratio; RBG, random blood; composite outcome risk - MV, admission in ICU and death; MV, mechanical ventilation, ICU - intensive care unit; HG, hyperglycemia; DM, diabetes mellitus; IFG, impaired fasting glucose; N/A, not available. * - adjusted for age, sex, smoking, systolic blood pressure, total cholesterol, admission to ICU, usage of - antihypertensive drugs, lipid-lowering agents, invasive mechanical ventilation, glucose-lowering drugs before inpatients and during inpatients, and corticosteroids.

## Iatrogenic Hyperglycemia

Steroid-induced hyperglycemia is frequently encountered in hospitalized patients. Past researches have demonstrated that 53–70% of patients without diabetes develop steroid-induced hyperglycemia (114). A study performed in Australia documented that 70% (n=80) of non-diabetic hospitalized people had no less than one blood glucose measurement of ≥ 10 mmol/L (115). The utilization of glucocorticoids in the setting of COVID-19 infection, mainly following RECOVERY trial publication, has increased (116). It could also be associated with an increased risk of developing diabetes, primarily due to the delayed or blunted recovery of β-cell damage (90).

Although high dosage therapeutic regimens of corticosteroids are well-known to be associated with the onset and deterioration of diabetes, hyperglycemia is not included in the list of remdesivir side effects. However, the medical society has been alarmed about the potential role of remdesivir in increasing blood sugar levels (117, 118). Supposedly, such associations and the possible mechanisms behind them are needed to be clarified *via* further investigations. It is evident that those implications have to be proven and has to be legitimated whether they are contributed only by remdesevir or mainly by the SARS-CoV-2 infection itself.

## Post-Discharge Considerations

As the data regarding the exact mechanisms and epidemiology of new-onset diabetes related to SARS-CoV-2 infection is scarce, it is challenging to advise any particular recommendations for post-discharge. Stress hyperglycemia may be transient in some people, and it may revert to normoglycemia following COVID-19 recovery (101, 113). Thus, they may not be classified as having diabetes and may not require any glucose-altering therapy. However, we presume that all COVID-19 hyperglycemic patients will require follow-up at 1^st^ month and at intervals of 3-6 months during the first-year post-discharge to determine if the new-onset diabetes is permanent or transient. Nevertheless, we strongly suggest that medical personnel should consider the likelihood that the non-diabetic COVID-19 hyperglycemia might be a harbinger of new or unmasked diabetes.

Prospective studies following COVID-19 related hospitalization are scarce. A systematic review from 2016 concluded that at a 3-month follow-up, 18,8% of patients with in-hospital hyperglycemia were with newly diagnosed diabetes. However, its results could potentially be biased due to different definitions of stress hyperglycemia, heterogeneity of enrolled participants, follow-up intervals and lack of COVID-19 pandemic (119). Regarding SARS-CoV-2 infection, in particular, a Chinese collective showed an incidence of 3,3% of new-onset diabetes at 6 months follow-up (120). The data from a healthcare registry of the US Department of Veterans Affairs showed an increased frequency of new-onset diabetes 6 months after COVID-19 infection (121). Additionally, Ayoubkhani et al. (2021) analyzed data from 47,780 people discharged following hospital admission for COVID-19 and reported that 4.9% developed diabetes at a mean follow-up of 140 days (60).

Considering the above-mentioned points, we may presume that new-onset diabetes associated with SARS-CoV-2 infection is a new potential risk to be expected in the post-COVID period. Moreover, it allows us to observe these patients in the long term and conduct research studies that include epidemiological and interventional approaches. Additionally, the CoviDIAB Project has been started by leading diabetes professionals and is dedicated to establishing and managing a global registry of patients with COVID-19-related diabetes (27). Hence, additional international collaborative research programs are essential to elucidate the natural disease epidemiology of COVID-19 and its consequences concerning carbohydrates metabolism.

## Conclusion

In brief, the extensive results of all aforementioned researches suggest that hyperglycemia in COVID-19 infection is a complex phenomenon. On one side, it could be new-onset hyperglycemia without diabetes as a result of stress, SARS-CoV-2 infection itself or unmask/latent diabetes. Conversely, it could be, of course, due to a deterioration of pre-existent diabetes. Nevertheless, it should be considered that the new-onset hyperglycemia without diabetes is associated with a poorer outcome and substantially higher rates of complications and overall mortality compared to normoglycemic individuals and those with prior diabetes.

In summary, it seems that the new-onset hyperglycemia without diabetes increases the composite outcome risk nearly 6-fold and the mortality risk approximately 3-fold compared to people without carbohydrate disturbances. Additionally, the mortality risk is nearly two times higher in COVID-19 patients with new-onset hyperglycemia without diabetes vs those with preexistent diabetes. Finally, new-onset diabetes is presumed to increase mortality risk 4-10 fold in contrast to normoglycemic patients. Worse outcomes were observed 2-4 times more frequently in patients with new-onset diabetes than those with pre-existent diabetes. It is worth, noting that there exists a significant inter-study variability of the aforementioned results.

So far, the available data is insufficient to clarify whether COVID-19 infection and associated stress hyperglycemia have any specificity with age, sex, ethnicity, and socioeconomic profile. To the best of our knowledge, only Coppelli et al. (2020) demonstrated that hyperglycemia, not DM remained a mortality predictor with an independent role for age and male sex. Additionally, Mamtani et al. (2020) demonstrated adjusted OR for hyperglycemia and age of 1.05 (21). In most other cases, studies have adjusted their results to age, sex, etc., but did not assess specificity for COVID-19 and stress hyperglycemia Further, prospective studies with larger cohorts are required to elucidate the pathophysiological mechanisms behind hyperglycemia in COVID-19 patients fully and to clarify whether hyperglycemia is a consequence or a causal primary factor.

## Author Contributions

AG - data gathering and manuscript preparation. YA - data gathering, manuscript preparation, and review and editing. ZK - data gathering, manuscript preparation, and review and editing. All authors contributed to the article and approved the submitted version.

## Conflict of Interest

The authors declare that the research was conducted in the absence of any commercial or financial relationships that could be construed as a potential conflict of interest.

## Publisher’s Note

All claims expressed in this article are solely those of the authors and do not necessarily represent those of their affiliated organizations, or those of the publisher, the editors and the reviewers. Any product that may be evaluated in this article, or claim that may be made by its manufacturer, is not guaranteed or endorsed by the publisher.
